# Genotoxic Properties of Synthetic Cannabinoids on TK6 Human Cells by Flow Cytometry

**DOI:** 10.3390/ijms21031150

**Published:** 2020-02-09

**Authors:** Monia Lenzi, Veronica Cocchi, Luca Cavazza, Sabrine Bilel, Patrizia Hrelia, Matteo Marti

**Affiliations:** 1 Department of Pharmacy and Biotechnology, Alma Mater Studiorum University of Bologna, 40126 Bologna, Italy; veronica.cocchi4@unibo.it (V.C.); patrizia.hrelia@unibo.it (P.H.); 2 Department of Morphology, Experimental Medicine and Surgery, Section of Legal Medicine and LTTA Center, University of Ferrara, 44121 Ferrara, Italy; luca.cavazza@student.unife.it (L.C.); sabrine.bilel@unife.it (S.B.); matteo.marti@unife.it (M.M.); 3 Collaborative Center for the Italian National Early Warning System, Department of Anti-Drug Policies, Presidency of the Council of Ministers, 44121 Ferrara, Italy

**Keywords:** synthetic cannabinoids, 5F-AKB-48, APINAC, STS-135, JWH-018-CL, JWH-018, BB-22, genotoxicity, mutagenicity, flow cytometry

## Abstract

Novel Psychoactive Substances (NPS) include several classes of substances such as synthetic cannabinoids (SCBs), an emerging alternative to marijuana, easily purchasable on internet. SCBs are more dangerous than Δ^9^-Tetrahydrocannabinol as a consequence of their stronger affinities for the CB_1_ and CB_2_ receptors, which may result in longer duration of distinct effects, greater potency, and toxicity. The information on SCBs cytotoxicity, genotoxicity, mutagenicity, and long-term effects is scarce. This fact suggests the urgent need to increase available data and to investigate if some SCBs have an impact on the stability of genetic material. Therefore, the aim of the present study was the evaluation of the mutagenic effect of different SCBs belonging to indole- and indazole-structures. The analyzes were conducted in vitro on human TK6 cells and mutagenicity were measured as micronucleus fold increase by flow cytometry. Our results have highlighted, for the first time, the mutagenic capacity of four SCBs, in particular in terms of chromosomal damage induction. We underline the serious potential toxicity of SCBs that suggests the need to proceed with the studies of other different synthetic compounds. Moreover, we identified a method that allows a rapid but effective screening of NPS placed on the market increasingly faster.

## 1. Introduction

The production and use of novel psychoactive substances (NPS) are constantly increasing worldwide and, particularly in Europe, the control of psychoactive substances has become a primary concern of governance and citizens. This is not surprising given the fact that NPS cause acute and chronic diseases with a significant cost to society, and they are also responsible for a large number of deaths. For example, in 2016, there were almost 8000 deaths due to this problem, excluding mortality due to long-term effects [1]. The acronym NPS includes several classes of substances such as cathinones, phenethylamine, synthetic opioids, and synthetic cannabinoids (SCBs).

In Europe, the first SCBs were identified in 2008 in various vegetable mixtures, called “herbal mixture” or “herbal blend”, and sold as incense or air fresheners. SCBs are an emerging alternative to marijuana, easily purchasable on internet as “Spice” and “not for human use”. Despite what has been stated on the labels, these products are capable to induce in the consumers stronger effects compared to those induced by cannabis [2]. From analyses conducted on “herbal mixture” products, carried out by different laboratories, the first SCBs identified in Italy were those of the JWH-like class (i.e., JWH-018, JWH-073, JWH-122, JWH-200, JWH- 250, JWH-251, JWH-081, JWH-398, JWH-019, and AM-2201) [3,4]. These were followed over the years by other SCBs with different chemical structures such as HU-210, CP 47, 497-C8 (including its analogues with C6, C8, and C9 alkyl chains), and others of the new generation, (i.e., 5F-AKB-48 and MDMB-CHMICA) [5,6]. These molecules act as full agonists on the CB_1_ cannabinoid receptors with an affinity of 10 to 100 times higher than Δ^9^-Tetrahydrocannabinol (Δ^9^-THC), which is a partial agonist. These pharmacodynamic properties demonstrate that synthetic compounds are even more dangerous than Δ^9^-THC [7]. Additionally, SCBs metabolites have stronger affinities for the CB_1_ and CB_2_ receptors compared to Δ^9^-THC, which may result in distinct effects [8]. Continued binding of metabolites to the CB_1_ receptor may be responsible for the greater potency and longer duration of pharmacologic effects as well as toxicity [9,10].

In addition to the emerging problem represented by the main adverse effects (i.e., agitation, anxiety, psychosis, drowsiness, tachycardia, hypertension, nausea, and vomiting), it is important to underline other completely atypical effects such as seizures, fever, stroke, myocardial infarction, shock, rhabdomyolysis, acute kidney injury, multiple organ failure, and death [11,12,13], as well as the consequent difficulty in managing the acute toxicity [14].

The SCBs pharmacological effects have been investigated by several groups in vitro and in vivo [15,16,17,18,19,20,21,22,23,24,25,26,27], but information on their cytotoxic, genotoxic, mutagenic, and long-term toxic properties is limited. However, one aspect of great toxicological and social importance, is represented by the potential mutagenicity induced by exposure to SCBs. In fact, the evaluation of the xenobiotic toxicological profile, in particular in terms of its genotoxic and mutagenic effects, is crucial to protect human health. Genotoxic substances are capable of interacting with the genetic material and causing an alteration that may be repaired with no consequences, induce apoptosis, or lead to a mutation that represents the fixation of the irreversible genetic damage transmissible to the progeny. Therefore, mutagenic substances are genotoxic [28].

The only results available indicate that the binding to receptors not only causes psychotropic effects, but also provokes pro-inflammatory response like Reactive Oxygen Species (ROS)-production, oxidative damage, and cell death [23,29]. Then, it is possible that SCBs cause toxic effects in users as a consequence of their impact on DNA-stability. For this reason, Koller et al. first investigated the genotoxic properties of some JWH-like drugs and obtained positive results in Single-Cell Gel Electrophoresis (SCGE) assay [30]. Subsequently the same group observed by Bacterial Reverse Mutation assay and Cytokinesis-Block Micronucleus (CBMN) test, that CP-47, 497-C8, and other SCBs induce DNA-damage at the chromosomal level while they do not cause gene mutations [31,32].

**Figure 1:**
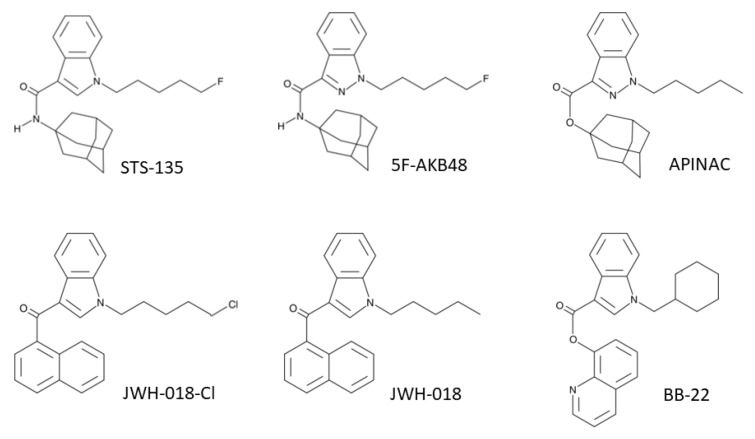
Chemical structures of STS-135, 5F-AKB-48, APINAC, JWH-018-Cl, JWH-018, and BB-22.

These findings suggest the urgent need to increase data available and investigate if some SCBs currently on the market have an impact on the stability of genetic material. For this reason, we decided to evaluate the potential genotoxic properties, in terms of mutagenicity, of six SCBs belonging to different indole- and indazole-SCBs structures. In particular, we tested: STS-135 [*N*-(Adamantan-1-yl)-1-(5-fluoropentyl)-1*H*-indole-3-carboxamide], JWH-018-Cl [1-(5-chloro-pentyl)-3-(1-naphthoyl)indole], JWH-018 [1-pentyl-3-(1-naphthoyl)indole], BB-22 [Quinolin-8-yl 1-(cyclohexylmethyl)-1*H*-indole-3-carboxylate], 5F-AKB-48 [*N*-(1-adamantyl)-1-(5-fluoropentyl)-1*H*-indazole-3-carboxamide], and APINAC [1-adamantyl 1-pentylindazole-3-carboxylate] (Figure 1).

For this purpose, it was necessary to select a suitable mutagenesis test and a proper assay system. Among those validated by the Organization of Economic Cooperation and Development (OECD), we identified the “In Vitro Mammalian Cell Micronucleus Test” as the micronucleus (MN) represents a sensitive biomarker of mutagenicity, in terms of chromosomal damage [33]. MN results from acentric chromosome break (induced by clastogen agents), which does not segregate correctly during cell division, or from whole chromosome (induced by aneuploidogen agents), that is unable to migrate to the opposite poles of the mitotic spindle during the anaphase. Subsequently, a nuclear membrane is formed generating a little additional nucleus that remains excluded from the main nuclei of daughter cells and that is easily identifiable by optical microscopy [34].

The corresponding OECD guideline n°487 reports, over the peripheral blood lymphocytes (PBL), several cell lines that can be used as validated test system (CHO, V79, CHL/IU, L5178Y and TK6) [33]. Among these, in particular, we selected the TK6 cells; SCBs were treated for 26 h, corresponding to the time required to carry out two replication cycle and thus transmit any eventual genetic damage to the progeny. This cell line is not of tumor origin and therefore is not characterized by the known instability of karyotype and p53 status mutation. Moreover, these cells are of human origin and so more representative of the possible effects in SCBs users, and finally, of lymphoblastoid type, that grow in suspension and so are particularly suitable for flow cytometry (FCM). This platform represents a valid alternative to optical microscopy [35], affected by some typical critical issues, such as the subjective reading, the long analysis times, and the number of cells analyzed, which is small to allow an appropriate and robust statistical analysis. To overcome these problems, we recently published a new automated protocol of the “In Vitro Mammalian Cell Micronucleus Test” by FCM [34], which we used in this study, for the evaluation of the possible SCBs mutagenicity.

## 2. Results

The research began with the preliminary determination of the concentrations to be used in subsequent experiments aimed at evaluating the potential mutagenicity of different SCBs. First, we evaluated the STS-135, 5F-AKB-48, APINAC, JWH-018-Cl, JWH-018, BB-22 induced cytotoxicity after 26 h treatment (corresponding to the time necessary for TK6 cells to carry out two cell cycle) by measuring the percentage of live cells at the different concentrations tested (12.5, 25, 35, 50, 75 µM). This value was normalized on that obtained in the untreated control cultures and made it possible to check for each SCB that the cellular viability respected the OECD threshold. Indeed, according to the OECD guidelines n°487, to assess the genotoxicity of a xenobiotic, the highest concentration tested should not induce a cytotoxicity, at most, equal to 55 ± 5% [33].

**Figure 2:**
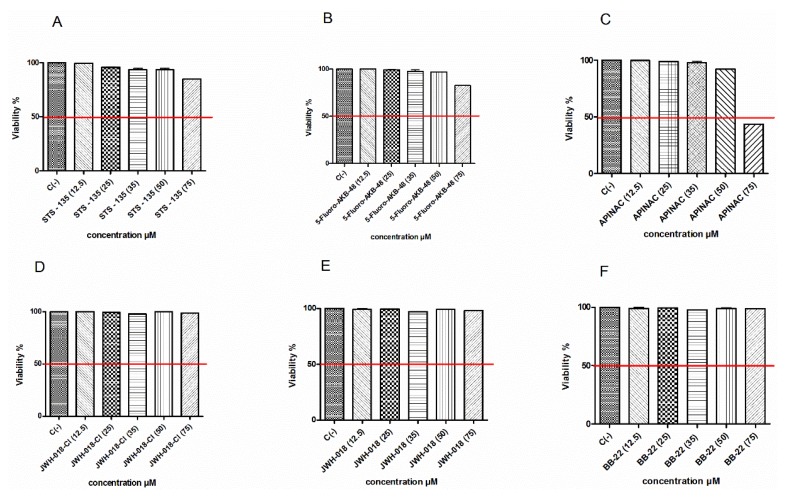
Cell viability on TK6 cells after 26 h treatment with STS-135 (**A**), 5F-AKB-48 (**B**), APINAC (**C**), JWH-018-Cl (**D**), JWH-018 (**E**), anf BB-22 (**F**) at the indicated concentrations with respect to the untreated control [C (-)]. Each bar represents the mean ± SEM of five independent experiments.

Figure 2 shows that the viability remains abundantly higher than the threshold required by the OECD (evidenced by the red line) for all substances up to 75 µM (Figure 2A,B,D–F), except for the APINAC (Figure 2C).

Furthermore, to make the analysis of mutagenesis reliable, it is necessary to measure cell proliferation to ensure that sufficient treated cells have undergone mitosis during the test and that the treatments are conducted at appropriate levels of cytoxicity also in terms of cytostasis. For this reason, the guideline n°487 states that: “is necessary to demonstrate that the cell in culture have divided, so that a substantial proportion of the cells scored have undergone division during or following a treatment time equal to 1.5–2 normal cell cycle lengths”. For this purpose, analogously to the cytotoxicity, the OECD establishes a threshold, at most, equal to 55 ± 5% and recommends the measurement of the Relative Population Doubling (RPD), to also estimate the cytostasis. For this reason, in parallel to cytotoxicity, the cytostasis was checked, in order to respect the threshold established by OECD [33]. Similarly, as previously demonstrated, all substances showed an RPD abundantly above to 55 ± 5% up to 75 µM concentration with the exception of APINAC (Table 1).

In paragraph 27, the guideline n°487 also suggests: “Assessment of other markers for cytotoxicity (e.g., cell integrity, apoptosis, necrosis…) could provide useful additional information” [33]. Therefore, the research continued by considering the possible apoptosis induction, on the one hand as alternative cell death mechanism, and on the other to avoid false positive results and to achieve a complete and correct definition of the SCBs concentrations to be tested for the mutagenesis evaluation. Indeed, the Guava ViaCount test, used for the cytotoxicity determination, could overestimate the percentage of live cells, based solely on the different membrane integrity, that characterize the live cells and necrotic cells and that are responsible for the different Propidium Iodide (PI) permeability. However, apoptotic cells, above in the early- to mid-stages, are characterized by a still intact membrane and so they are non-permeant to the dye. This fact could lead one to mistakenly consider the apoptotic cells as viable cells.

Moreover, it is necessary to underline that the selected protocol of mutagenesis analysis by FCM could lead to the possible confounding by the instrument, of apoptotic bodies with MN being both made up of chromatin and therefore highlighted by SYTOX Green staining.

**Figure 3:**
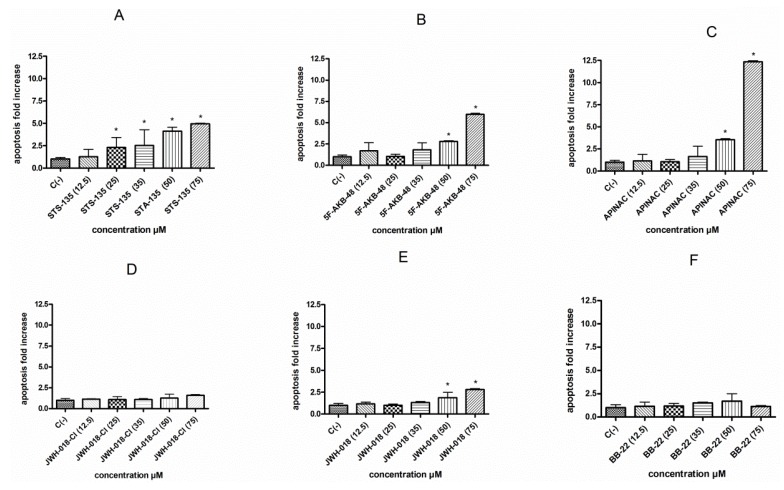
Apoptosis fold increase on TK6 cells after 26 h treatment with STS-135 (**A**), 5F-AKB-48 (**B**), APINAC (**C**), JWH-018-Cl (**D**), JWH-018 (**E**), and BB-22 (**F**) at the indicated concentrations with respect to the untreated control [C (-)]. Each bar represents the mean ± SEM of five independent experiments. Data were analysed using repeated ANOVA followed by Dunnet post-test. * *p* < 0.05 versus C (-).

The involvement of the apoptotic process was analyzed by the Guava Nexin assay, which allowed us to highlight a different behavior among the six SCBs tested. In particular, the STS-135 was shown to induce a statistically significant apoptosis fold increase at all concentrations tested except at 12.5 µM. On the contrary, the cultures treated with 5F-AKB-48, APINAC, and JWH-018, showed a similar trend, corresponding to a statistically significant apoptosis fold increase starting from the 50 µM concentration. Finally, JWH-018-Cl and BB-22 were not found to induce apoptosis at any of the concentrations tested (Figure 3A–F).

In light of the results obtained from the cytotoxicity, cytostasis, and apoptosis analysis, the concentrations to be used for the evaluation of mutagenicity were selected. In particular, STS-135 12.5 and 25 µM, 5F-AKB-48 25 and 35 µM, APINAC 25 and 35 µM, JWH-018-Cl 35 and 50 µM, JWH-018 25 and 35 µM, and BB-22 35 and 50 µM concentrations were tested, because overall they were abundantly below the cytotoxicity and cytostasis OECD threshold and did not induce apoptosis or, at most, they caused a 2-fold increase in apoptosis.

**Figure 4:**
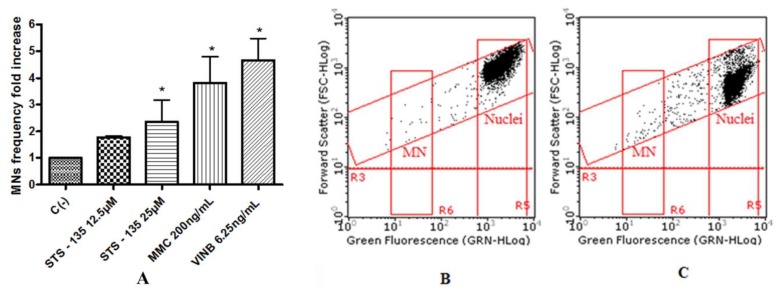
MN’s frequency fold increase on TK6 cells after 26 h treatment with STS-135 at the indicated concentrations with respect to the untreated negative control [C (-)] and to positive controls [MMC and VINB] (**A**), plot of nuclei and micronuclei in untreated control (**B**), and in 25µM treated (**C**). Data were analyzed using repeated ANOVA followed by Bonferroni post-test. * *p* < 0.05 versus C (-).

**Figure 5:**
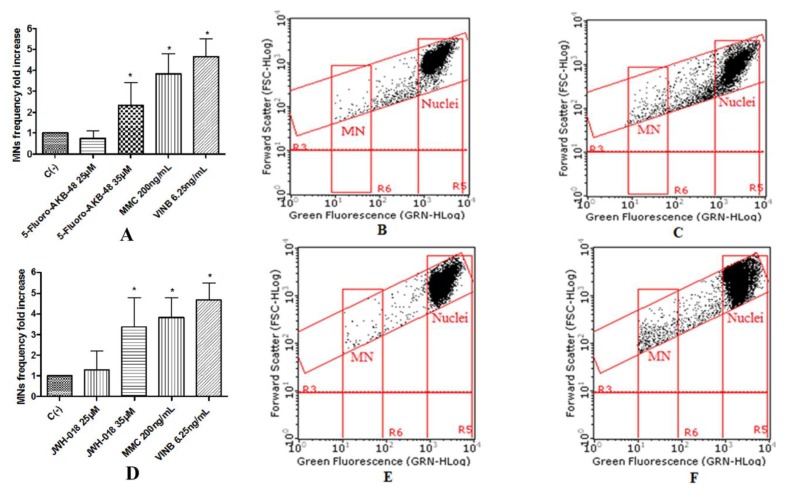
MN’s frequency fold increase on TK6 cells after 26 h treatment with 5F-AKB-48 (**A**) and JWH-018 (**D**) at the indicated concentrations with respect to the untreated negative control [C (-)] and to positive controls [MMC and VINB], plot of nuclei and micronuclei in untreated control (**B**,**E**) and in 35 µM treated (**C**,**F**). Data were analyzed using repeated ANOVA followed by Bonferroni post-test. * *p* < 0.05 versus C (-).

**Figure 6:**
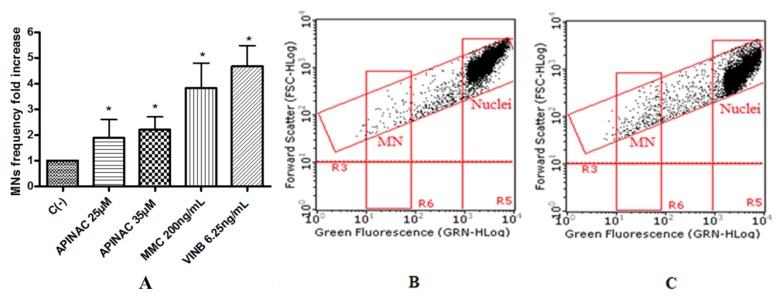
MN’s frequency fold increase on TK6 cells after 26 h treatment with APINAC at the indicated concentrations with respect to the untreated negative control [C (-)] and to positive controls [MMC and VINB] (**A**), plot of nuclei and micronuclei in untreated control (**B**) and in 25 µM treated (**C**). Data were analyzed using repeated ANOVA followed by Bonferroni post-test. * *p* < 0.05 versus C (-).

**Figure 7:**
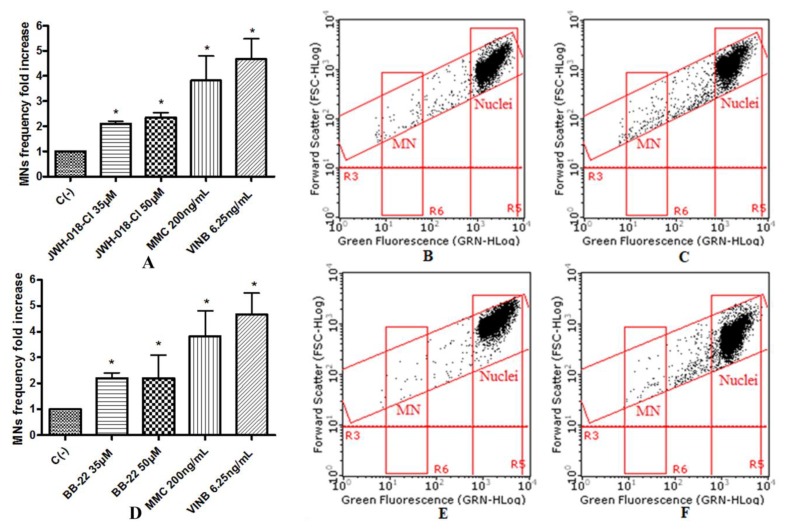
MN’s frequency fold increase on TK6 cells after 26 h treatment with JWH-018-Cl (**A**) and BB-22 (**D**) at the indicated concentrations with respect to the untreated negative control [C (-)] and to positive controls [MMC and VINB], plot of nuclei and micronuclei in untreated control (**B**,**E**) and in 35 µM treated (**C**,**F**). Data were analyzed using repeated ANOVA followed by Bonferroni post-test. * *p* < 0.05 versus C (-).

To assess the potential mutagenic effect induced by the SCBs, the frequency of MNs was measured in untreated negative control cultures, SCBs-treated cultures, and positive control cultures [Mytomicin (MMC) or Vinblastine (VINB) treated]. All SCBs resulted as able to cause mutagenicity as confirmed by the MNs frequency fold increase. Especially, as one can observe in the plot in Figure 4, the STS-135 increased, in a statistically significant manner, the MNs frequency at the 25 µM concentration. A similar behavior was recorded for 5F-AKB-48 and JWH-018 at 35 µM concentration (Figure 5), while for the APINAC, both the concentrations tested (25 µM and 35 µM) showed mutagenic capacity (Figure 6), likewise to the JWH-018-Cl and the BB-22 (35 µM and 50 µM) (Figure 7).

## 3. Discussion

The neuropharmacological, neurochemical, and behavioral effects of various substances belonging to the NPS category are well known and widely demonstrated [7,36,37]. Also, with regard to SCBs, recent studies are available aimed at investigating, mainly in vivo, the psychostimulant and neuropharmaco-toxicological effects [23,24,26]. On the contrary, according to our knowledge, very little information is currently available about genotoxicity, mutagenicity, and the consequent potential long-term effects of SCBs. For example, bibliographic research, conducted on the main databases (i.e., Scopus from Elsevier and PubMed from MEDLINE), allowed us to identify only one publication on JWH-018 [30] and a publication on 5F-AKB-48 [32], while there are no studies on the impact on DNA following treatment with STS-135, APINAC, JWH-018-Cl, and BB-22.

In order to protect human health, it is fundamental to evaluate the safety of all substances and the risk associated with the exposure, in particular in terms of the effects on genetic material, to exclude genotoxicity and mutagenicity. Indeed, the introduction of Registration, Evaluation, Authorization, and restriction of Chemicals (REACH) required the study of the toxicity of many chemicals already on the market, providing the priority of the mutagenic potential evaluation. Consequently, it is extremely necessary to investigate this aspect also for drugs of abuse by protocols, which allow a rapid and accurate screening.

The tests currently validated by the OECD are distinguished on the basis of the type of damage they allow one to highlight (gene mutation, structural chromosomal aberration, and aneuploidy) and on the fact that some can be conducted in vitro, while others require the use of animal testing [38].

On the other hand, increasing attention, both from public opinion and from the scientific community, is aimed at avoiding animal tests, deemed not strictly necessary, and some regulatory genetic toxicology test strategies encourage the use of in vitro tests. The REACH specifically requires the execution of mutagenesis tests exclusively in vitro, in line with the 3R principle, introduced in 1959 by Russel and Burch, in order to promote the alternative methods, i.e., all the procedures useful to reduce the use of animals (Reduction), to completely replace them (Replacement), but also to limit or eliminate their suffering by refining experimental conditions (Refinement).

Therefore, in the present study, we evaluated the mutagenic effect of six SCBs belonging to different indole- and indazole-SCBs structures by the “In Vitro Mammalian Cell Micronucleus Test” on TK6 cell line. The fact that SCBs are psychoactive substances could suggest testing their effects on nervous system, but it should be emphasized that the OECD guideline 487 consider several cell lines validated among which nervous system cells are not included and that the standard in vitro mutagenesis evaluation does not provide the ability to select the cell type on the basis of the different substance under study, but on the basis of cellular characteristics well defined, such as the ability to grow well in culture, the stability of karyotype, and spontaneous MNs frequency [33].

Our results enabled us, for the first time, to highlight the mutagenic capacity of STS-135, APINAC, JWH-018-Cl, and BB-22 and confirmed what was previously demonstrated by Koller et al., first for JWH-018 then for 5F-AKB-48. In particular, the Guava ViaCount assay showed that all the substances under study do not induce cytotoxicity and cytostasis at all concentrations tested, except for APINAC at 75 µM concentration. These results agree with those previously published on tumor cell lines by Koller et al. [30,32] and Couceiro et al. [39], and the absence of cytotoxicity could be considered a positive result, but in reality, it is not at all from the point of view of genotoxicity. They are indeed particularly alarming data, considering that a substance capable of damaging DNA, but which allows the cell population to survive and replicate, also makes it able to transmit any genetic damage to offspring. For these reasons, the OECD guideline n°487 recommends proceeding with the evaluation of genotoxicity, only if the treated population shows a viability and a cell proliferation of at least 40% when compared to untreated control cultures [33].

However, the Guava ViaCount assay does not allow one to highlight apoptosis induction. In fact, it is simply based on the different membrane integrity between viable cells and necrotic cells and consequent permeability to the dye. Therefore, apoptotic cells, characterized by a stil-intact membrane, could be confused by the instrument as viable cells. We therefore considered it necessary to proceed with a more specific test to highlight this alternative death mechanism, again particularly important for genotoxicity. In fact, on the one hand, the cell population exposed to a xenobiotic could be stimulated to undergo apoptosis, following unrepaired genetic damage, while on the other hand, the cytofluorimetric method used could lead to false positives, due to the possible confounding between apoptotic bodies and MNs by the instrument [34]. The results obtained with the Guava Nexin assay allowed us to highlight a different behavior for the different substances analyzed. In fact, STS-135 stimulated apoptosis of TK6 cells, at all tested concentrations equal to or greater than 25 µM, 5F-AKB-48, APINAC, and JWH-018 only starting from 50 µM concentration, while JWH-018-Cl and BB-22 did not increase the percentage of apoptotic cells, with significant consequences in terms of mutagenicity, because it highlights the cell’s inability to counteract, through this mechanism of selective death, the transmission of genetic damage immediately to the daughter cells. Overall, based on the results obtained, the concentrations to be used for the evaluation of mutagenicity were selected.

The automated cytofluorimetric protocol published by Lenzi et al. [34] demonstrates the ability to statistically significantly increase the MNs frequency for all substances and thus prove mutagenic, in terms of its ability to induce chromosomal aberrations. In particular, APINAC, JWH-018-Cl, and BB-22 have shown their mutagenicity at both concentrations tested. 5F-AKB-48 behavior confirmed what is reported by Koller et al., both in terms of cytotoxicity and cystostasis, and in terms of MNs frequency [32]. In fact, in this case, an increase of two and three times was also shown at 50 and 75 µM concentrations, respectively; in our case, however, these results were not shown, because the concentrations were excluded, on the basis of the apoptosis test. 5F-AKB-48, similarly to JWH-018, statistically increased the frequency of MNs significantly, but only at the highest tested concentration (35 µM); yet, these data do not have great importance in light of the impossibility for genotoxic substances to define a No-Observed-Adverse-Effect Level (NOAEL). By simply increasing the dose and exposure, the likelihood that damage will occur increases [28].

Similarly, a comparison between the different increases induced by the different SCBs does not have a particular toxicological significance. In fact, on the one hand, it could allow one to distinguish the strong- and weak-genotoxic agents, while on the other hand, the fundamental result for the risk assessment is the positive or negative response.

Still, in relation to the doses tested in our in vitro study, it could be asked whether they are reachable in vivo and comparable to the level of SCBs detectable in the human serum upon intake. However, it is important to underline that, given the lipophilic characteristics of the SCBs studied, it is easy to hypothesize an accumulation, especially at the level of the upper airway epithelial cells, following inhalation or intake with tobacco smoke [40]. Moreover, once again, it should be reiterated that it is recognized that for genotoxic substances zero risk corresponds only to zero dose, and therefore, any dose is potentially toxic. In addition, as far as the evaluation of mutagenicity is concerned, the OECD recognizes the data obtained in vitro sufficient in case of positivity (obviously if they have been achieved through validated assay and test systems recognized) [38].

The induction of apoptosis, demonstrated in some cases only at higher concentrations, could therefore be confirmed as a consequence of the genetic damage, that it was possible to highlight already at the lower doses, but evidently sub-apoptotic and therefore, in a certain sense even more dangerous.

In the study by Koller et al., the S9 mix was used as an extrinsic metabolic activation in the Ames assay, in order to highlight whether the metabolites were able to induce gene mutations, but on the basis that the parent compounds had not shown this capacity [32]. In our case, on the other hand, all substances tested are mutagenic by themselves and therefore, we have not considered it necessary to investigate the activity of any metabolites. However, the research will also continue in this direction, for a more complete definition of the mechanism underlying the further serious potential toxicity to be demonstrated.

Finally, the cytofluorimetric method, used for the first time to evaluate the genotoxicity of SCBs, offers numerous advantages compared to the classical method, since by analyzing a number of events 10 times higher, it allows robust statistical analysis and also identifies the so-called weak genotoxic. It guarantees a much more objective result, that is not affected by the subjectivity of operator interpretation, as it happens in microscopy, with a considerable reduction in analysis times [34], which allows for a much faster screening of the effects induced by the NPS, increasingly faster placed on the market.

## 4. Materials and Methods

### 4.1. Reagents

Ethylenediaminetetraacetic acid [EDTA], Fetal Bovine Serum (FBS), L-Glutamine (L-GLU), Nonidet, Penicillin-Streptomycin solution (PS), Potassium Chloride, Potassium Dihydrogen Phosphate, Roswell Park Memorial Institute (RPMI) 1640 medium, Water bpc grade, Ethanol, Sodium Chloride, Sodium Hydrogen Phosphate (all purchased from Sigma-Aldrich, St Louis, Missouri, USA), Guava Nexin Reagent, Guava ViaCount Reagent (all purchased from Luminex Corporation, Austin, Texas, USA), RNase A, SYTOX Green, 7-aminoactinomycinD (7-AAD) (purchased from Thermo Fisher Scientific, Waltham, Massachusetts, USA).

### 4.2. Synthetic Cannabinoid Compounds

The SCBs STS-135, 5F-AKB-48, APINAC, JWH-018-Cl, JWH-018, and BB-22 were purchased from LGC Standards (LGC Standards S.r.L., Sesto San Giovanni, Milan, Italy) and www.chemicalservices.net.

All synthetic cannabinoids were dissolved in absolute ethanol up to 10 mM stock solution and stored at –20 °C. Absolute ethanol concentration was always in the range 0.25–0.75% in all experimental conditions, to avoid potential solvent toxicity.

### 4.3. Cell Culture and Treatments

Human TK6 lymphoblast cells were purchased by Sigma-Aldrich (St. Louis, Missouri, USA) and were grown at 37 °C and 5% CO_2_ in RPMI-1640 supplemented with 10% FBS, 1% L-GLU and 1% PS. To maintain exponential growth and considering that the time required to complete the cell cycle is 13 h, the cultures were divided every third day in fresh medium and the cell density did not exceed the critical value of 9 × 10^5^ cells/mL.

In all experiment, aliquot of 2 × 10^5^ of TK6 cells were treated with increasing concentrations of SCBs from 0 to 75 µM and incubated for 26 h, corresponding to two replication cycles.

### 4.4. Flow Cytometry

All FCM analysis reported below were performed using a Guava easyCyte 5HT flow cytometer equipped with a class IIIb laser operating at 488 nm (Merck, Darmstadt, Germany).

### 4.5. Cytotoxicity Analysis

**Figure 8:**
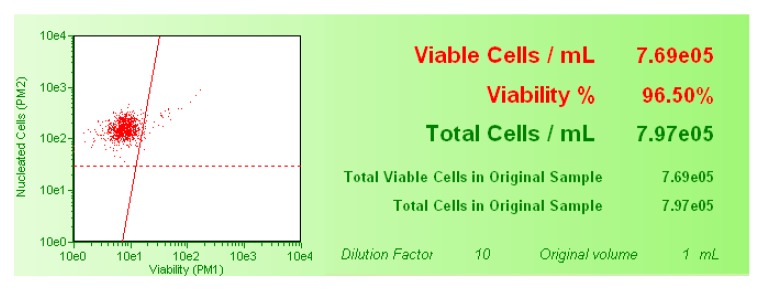
Example of the two populations identifiable with the Guava ViaCount Reagent and analyzed by the Guava ViaCount software. On the right are the necrotic cells PI positive and on the left are the live cells PI negative.

In order to detect cytotoxicity, the percentage of viable cells was evaluated by the Guava ViaCount Assay. After SCBs treatment, Guava ViaCount Reagent was added to the cells to discriminate viable from dead cells. This reagent contains the dye PI, which is only able to penetrate the altered membrane of necrotic cells, bind covalently to the DNA, and emit red fluorescence. In contrast, cells with an integral membrane are impermeable to PI and, thus, emit low red fluorescence. The viability and the total viable cell number were calculated automatically by the Guava ViaCount software (Merck, Darmstadt, Germany) and shown in the box next to the dot plot (Figure 8).

The viability percentage recorded in the cultures treated with SCBs different concentrations were normalized on those recorded in the untreated control cultures, equal to 100%. These results were used to check for each SCB that the cellular viability percentage respected the OECD threshold [33].

### 4.6. Cytostasis Analysis

At the same time, the Guava ViaCount assay also allowed, as previously explained, to count the number of cells seeded at time 0 (initial cell number) and that measured at the end of the treatment time (post-treatment cell number). From these values was derived the Population Doubling (PD):

(1) PD=[log (Post−treatment cell number ÷Initial cell number)]÷log2

Subsequently, the PD obtained in the control cultures was compared to that measured in the treated cultures, obtaining the RPD value, to measure the cell proliferation and to check that a substantial proportion of the cells have undergone division at the end of treatment time [33].

(2) $$\mathrm{RPD}\;=\;\;\frac{\left(No.\;\;of\;Population\;doublings\;in\;treated\;cultures\right)}{\left(No.\;\;of\;Population\;doublings\;in\;control\;cultures\right)}\times 100$$

### 4.7. Apoptosis Analysis

**Figure 9:**
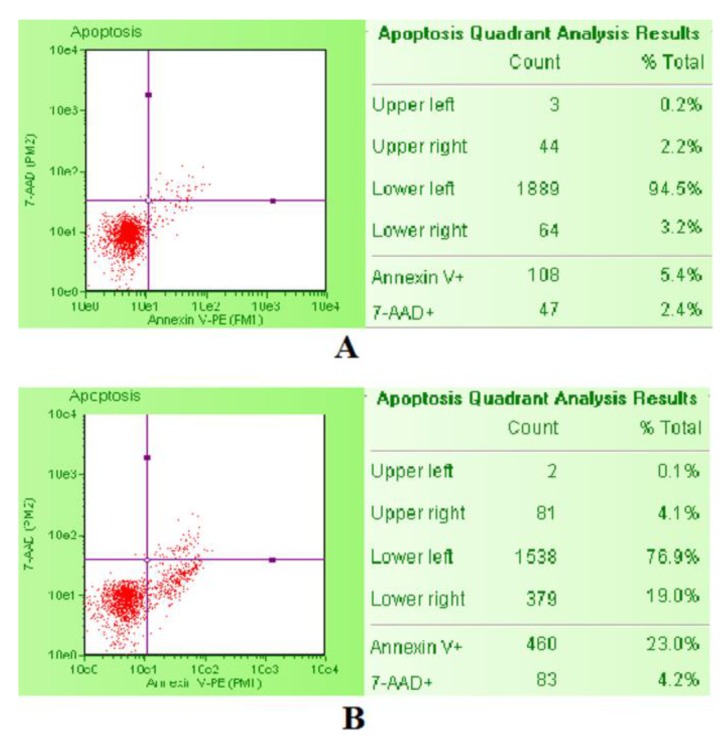
Example of the three populations identifiable with the Guava Nexin Reagent and analyzed with the Guava Nexin Software in untreated negative control (**A**) and staurosporine-treated positive control (**B**). On the lower left are the live cells 7-AAD and Annexin-V-PE negative, on the lower right the apoptotic cells 7-AAD negative and Annexin-V-PE positive, on the upper right the necrotic cells 7-AAD and Annexin-V-PE positive.

The apoptosis induction was evaluated by the Guava Nexin Assay. After SCBs treatment, Guava Nexin Reagent was added to the cells. The reagent contained two dyes, 7-aminoactinomycin D (7-AAD) and Annexin-V-PE. As previously described for PI, 7-AAD allows the discrimination between live and dead cells, while Annexin-V-PE allows identification of apoptotic cells by binding to phosphatidylserine and emitting yellow fluorescence. In particular, viable cells are 7-AAD and Annexin-V-PE negative, apoptotic cells are 7-AAD negative and Annexin-V-PE positive, and necrotic cells are 7-AAD and Annexin-V-PE positive. We used staurosporine 1 µM as positive control, being a known apoptosis inducer. The comparison between the untreated negative control cultures and the staurosporine-treated positive control cultures by the previously described double coloring allow one to correctly position the gates. Then, the Guava Nexin software (Merck, Darmstadt, Germany) automatically calculated the percentage of viable, apoptotic, and necrotic cells and showed it in the box next to the dot plot (Figure 9).

The apoptotic cell percentages recorded in the cultures treated with different SCBs concentrations were normalized on those recorded in the untreated control cultures, equal to 1% and expressed as apoptotic fold increase. These results were used to check for each SCB that the apoptosis induction was similar or corresponding at most to a doubled increase of that recorded in the untreated control cultures [33].

### 4.8. Genotoxicity Analysis

**Figure 10:**
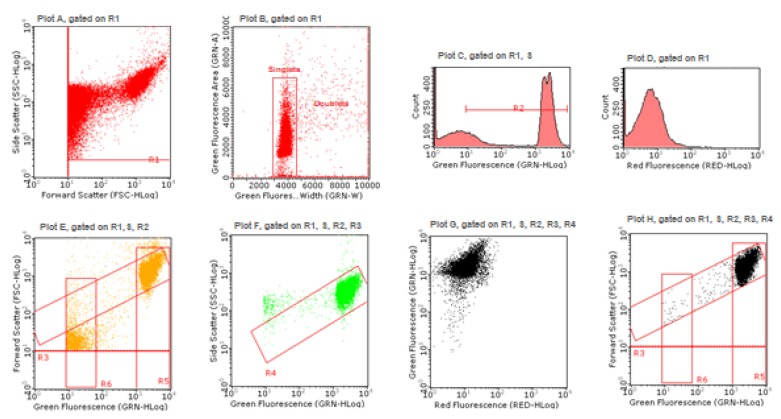
Gating strategy to analyze nuclei and MNs by Guava Incyte software (Merck, Darmstadt, Germany).

The analysis of the MNs frequency was performed by using an automated protocol published by Lenzi et al. [34] and using MMC and VINB as positive controls, since they are a well-known clastogen and aneuploidogen agent, respectively [33]. In particular, after 26 h of SCBs exposure, aliquots of 7 × 10^5^ cells were collected and first stained with 7-AAD, which allows the discrimination between viable and dead cells, being able only to penetrate the altered membrane of necrotic cells. Subsequently, the cells were treated with lysis solution containing SYTOX Green, able to label all chromatin. Therefore, this double staining allowed us to highlight nuclei and MNs deriving only from viable and proliferating cells. In Figure 10, the gating strategy is described and shown step by step (Figure 10).

The results were expressed as MNs frequency fold increase in SCBs-treated cultures compared to that present in the untreated control cultures.

In plot A, the gate R1 allows one to remove debris and to select the events to be considered, on the basis of Forward Scatter (FSC) versus Side Scatter (SSC); doublets discrimination occurs in plot B, using green fluorescence pulse processing (width versus area) and by the gate “singlets”; in plot C, the gate R2 selects, between 10^1^ and 10^4^, the range signal to be considered, on the basis of MNs presenting a green fluorescence of about 100 times smaller than nuclei, positioned between 10^3^ and 10^4^; in plot D, the red fluorescence PMT voltage adjusting allows one to visualize nuclei deriving from viable cells, in the low red fluorescence region and to discriminate them from nuclei deriving from dead cells, potentially present in the high red fluorescence region; first, the gate R3 (plot E) and after the gate R4 (plot F) allows one to remove cell lysis residues and to select the events to be considered on the basis of green fluorescence versus FSC and of green fluorescence versus SSC, respectively; the SYTOX Green /7-AAD correct compensation is checked in plot G and in the last plot H occurs the final discrimination between nuclei and MNs on the basis of the different SYTOX Green fluorescence intensity and of the different size analyzed by FSC, because the MNs size varies between 1/3rd and 1/16th of the mean size of the main nuclei. The gate R5 (nuclei) and the gate R6 (MN) allow the software to perform statistical analysis and to measure for each sample the MNs frequency on 10,000 nuclei.

### 4.9. Statistical Analysis

All results were expressed as mean ± SEM of at least five independent experiments. For the statistical analysis of the apoptosis and MNs, we used the Analysis of Variance for paired data (repeated ANOVA), followed by Dunnett or Bonferroni as post-test. All the statistical analyses were performed using Prism Software 4 (GraphPad Software, San Diego, CA, USA).

## 5. Conclusions

In this work, we underline the serious potential toxicity of SCBs, that suggests the need to proceed with the studies of other different synthetic compounds. Moreover, we identified a method which allows a rapid but effective screening of NPS, increasingly faster placed on the market.

## Figures and Tables

**Table 1:** Relative Population Doubling (RPD) on TK6 cells after 26 h treatment with STS-135, 5F-AKB-48, APINAC, JWH-018-Cl, JWH-018, and BB-22 at the indicated concentrations with respect to the untreated control [C (-)]. Data are presented as mean ± SEM of five independent experiments.

**STS-135**	**RPD**	**5F-AKB-48**	**RPD**	**APINAC**	**RPD**
C (-)	100.0%	C (-)	100.0%	C (-)	100.0%
12.5 µM	82.1% ± 0.1	12.5 µM	84.1% ± 0.3	12.5 µM	83.2% ± 0.1
25 µM	68.6% ± 0.2	25 µM	81.7% ± 0.1	25 µM	81.2% ± 0.3
35 µM	71.6% ± 0.1	35 µM	80.6% ± 0.2	35 µM	72.6% ± 0.3
50 µM	66.0% ± 0.3	50 µM	70.0% ± 0.2	50 µM	52.2% ± 0.2
75 µM	62.3% ± 0.2	75 µM	61.4% ± 0.1	75 µM	35.7% ± 0.4
**JWH-018-Cl**	**RPD**	**JWH-018**	**RPD**	**BB-22**	**RPD**
C (-)	100.0%	C (-)	100.0%	C (-)	100.0%
12.5 µM	95.9% ± 0.2	12.5 µM	90.1% ± 0.1	12.5 µM	82.4% ± 0.2
25 µM	94.4% ± 0.2	25 µM	89.4% ± 0.4	25 µM	78.8% ± 0.3
35 µM	86.1% ± 0.3	35 µM	75.5% ± 0.3	35 µM	81.1% ± 0.1
50 µM	88.2% ± 0.3	50 µM	82.4% ± 0.1	50 µM	86.4% ± 0.4
75 µM	81.6% ± 0.3	75 µM	75.5% ± 0.3	75 µM	79.8% ± 0.2

## Abbreviation

5F-AKB-48	*N*-(1-adamantyl)-1-(5-fluoropentyl)-1*H*-indazole-3- carboxamide
APINAC	1-adamantyl 1-pentylindazole-3-carboxylate
BB-22	Quinolin-8-yl 1-(cyclohexylmethyl)-1*H*-indole-3-carboxylate
CBMN	Cytokinesis-Block Micronucleus
FCM	Flow Cytometry
FCS	Forward Scatter
JWH-018	1-pentyl-3-(1-naphthoyl) indole
JWH-018-Cl	(1-(5-chloro-pentyl) -3-(1-naphthoyl) indole)
MMC	Mitomycin
MNs	Micronucleus
NOAEL	No-Observed-Adverse-Effect Level
NPS	New Psychoactive Substances
OECD	Organization of Economic Cooperation and Development
PBL	Peripheral Blood Lymphocytes
PI	Propidium Iodide
ROS	Reactive Oxygen Species
RPD	Relative Population Doubling
SCB	Synthetic Cannabinoids
SCGEs	Single Cell Gel Electrophoresis
STS-135	*N*-(Adamantan-1-yl)-1-(5-fluoropentyl)-1*H*-indole-3-carboxamide
Δ^9^-THC	Δ^9^-Tetrahydrocannabinol
VINB	Vinblastine
